# Leveraging CTSA hubs for rapid, large-scale, high-impact research: A case study during a global public health emergency

**DOI:** 10.1017/cts.2022.484

**Published:** 2022-10-18

**Authors:** Jennifer A. Croker, Shannon Valenti, Holly Ann Baus, Eric W. Ford, David Mathias, Laurel Yasko, Dan McGaughey, Tony Smith, Katherine Underwood, Jennifer Avolio, Kaitlyn Sadtler, Matthew J. Memoli, Robert P. Kimberly, Steven E. Reis

**Affiliations:** 1 Center for Clinical and Translational Science, University of Alabama at Birmingham, Birmingham, AL, USA; 2 Clinical and Translational Science Institute, University of Pittsburgh, Pittsburgh, PA, USA; 3 LID Clinical Studies Unit, Laboratory of Infectious Diseases, Division of Intramural Research, National Institute of Allergy and Infectious Diseases, National Institutes of Health, Bethesda, MD, USA; 4 Department of Health Care Organization, and Policy, School of Public Health, University of Alabama at Birmingham, Birmingham, AL, USA; 5 Section on Immuno-Engineering, National Institute of Biomedical Imaging and Bioengineering, National Institutes of Health, Bethesda, MD 20894, USA

**Keywords:** COVID-19, serosurvey, CTSA, remote study visit, diversity, rural populations, pandemic preparedness, team science

## Abstract

As the COVID-19 pandemic took hold in the USA in early 2020, it became clear that knowledge of the prevalence of antibodies to severe acute respiratory syndrome coronavirus 2 (SARS-CoV-2) among asymptomatic individuals could inform public health policy decisions and provide insight into the impact of the infection on vulnerable populations. Two Clinical and Translational Science Award (CTSA) Hubs and the National Institutes of Health (NIH) set forth to conduct a national seroprevalence survey to assess the infection’s rate of spread. This partnership was able to quickly design and launch the project by leveraging established research capacities, prior experiences in large-scale, multisite studies and a highly skilled workforce of CTSA hubs and unique experimental capabilities at the NIH to conduct a diverse prospective, longitudinal observational cohort of 11,382 participants who provided biospecimens and participant-reported health and behavior data. The study was completed in 16 months and benefitted from transdisciplinary teamwork, information technology innovations, multimodal communication strategies, and scientific partnership for rigor in design and analytic methods. The lessons learned by the rapid implementation and dissemination of this national study is valuable in guiding future multisite projects as well as preparation for other public health emergencies and pandemics.

## Introduction

On March 11, 2020, a pandemic was declared by the World Health Organization (WHO) due to a novel respiratory viral infection, the likes of which had not been witnessed in generations [1]. COVID-19, the illness caused by severe acute respiratory syndrome coronavirus 2 (SARS-CoV-2) caused a range of symptoms from totally undetectable (i.e., “asymptomatic”) to flu-like effects to severe health issues that necessitated emergency medical care, hospitalization, and intensive care. Two years after the first documented case in the USA [2], over 68 million individuals across the country had been diagnosed with COVID-19 and over 1 million deaths have been attributed to the virus. In the same time frame, case counts around the world approached half a billion, with over six million reported deaths [3,4]. One of the most significant early challenges in managing this outbreak was the lack of accurate viral prevalence and infection rates – especially among asymptomatic carriers.

Recognizing its pressing scientific and public health obligations, investigators at the National Institutes of Health (NIH) initiated a study to examine the presence of antibodies (seroprevalence) to SARS-CoV-2 in blood specimens collected from participants without a known diagnosis or symptoms of COVID-19. The study was initially designed to enroll a geographically constrained population that could physically visit the recruitment site on the NIH campus. Given the rapid spread of the disease, the NIH team soon realized that the study needed to scale across the country to have the greatest impact on the development of diagnostics that were reliable and generalizable and to inform national policies to mitigate the life-threatening impact of the pandemic in the USA. Therefore, they needed to expand the research sites and teams to other locations.

In 2006, the NIH Clinical and Translational Science Award (CTSAs) program established a consortium of research-intensive hubs to realize a vision for the biomedical research enterprise of the future. The program is empowered with the flexibility and support to innovate clinical and translational research workflows, develop critical scientific capacities and expertise, assemble a knowledgeable workforce, and engage contributors in an integrated approach to accelerating the translation of discoveries to improve health and health equity of individuals and communities [5,6]. This ethos uniquely positions National Center for Advancing Translational Sciences (NCATS)-supported CTSA sites to pivot and scale in response to scientific opportunity. By virtue of their established research capacities, expertise and experience in large, multisite projects, two CTSA hubs (University of Pittsburgh (Pitt CTSI) and University of Alabama at Birmingham (UAB CCTS)) proposed a collaboration with the NIH in April 2020 to rapidly expand their seroprevalence study to a national scale.

**Fig. 1.**
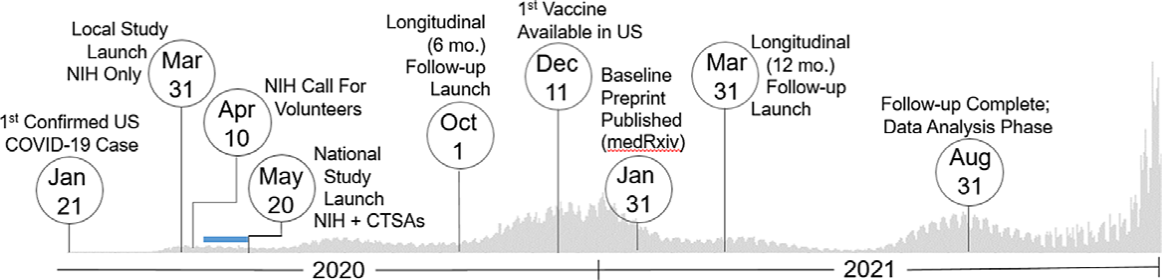
Study timeline. Set against the backdrop of actual US COVID-19 reported case counts [3,4], the collaborative team launched and regularly adapted an immune surveillance study across the nation that included three specimen and data collection time points (0, 6, and 12 months). The serosurvey project began as a single-site study based at the intramural NIH campus in March 2020. Over 400,000 persons nationally responded to an NIH press release calling for volunteers in mid-April 2020. As a result, the initiative quickly pivoted to a multisite study by leveraging two CTSA sites based at the University of Pittsburgh and the University of Alabama at Birmingham (UAB), which launched recruitment May 20, 2020. Over the next 15 months, the team succeeded to enroll over 10,000 individuals representative of the geographic and demographic diversity of the country. Blue Bar: Based on previously established capacity, trained workforce, and scientific agility, the two CTSA Hubs were quickly able to submit grants, establish IRB reliance, implement a comprehensive communications strategy, staff recruitment teams (during a pandemic work stoppage), receive and store 15000 kits, standardize a shipping protocol, and tailor informatics tools for rigorous data management (REDCap, Salesforce) to go live with recruitment ∼ 1 month later. Legend: CTSAs, Clinical and Translational Science Awards; mo., month; NIH, National Institutes of Health; US, United States.

The aim of the collaboration was to study COVID-19 positivity rates among asymptomatic individuals to inform national and local policy initiatives to combat the virus’ spread. The team recognized early on the critical need for having diverse representation in study participation to assess the potential impact of health disparities. By leveraging and pivoting existing resources at the CTSA hubs, the partnership succeeded in quickly developing and implementing comprehensive and harmonized processes that enabled recruitment of participants from all 50 states, data collection, specimen acquisition, and analyses (Fig. 1). Herein, the group details several lessons learned about creating a national research program, which may inform the conduct of future multisite investigation and pandemic preparedness.

## Methods

### Case Study: Undiagnosed SARS-CoV-2 Seropositivity in the USA

As the public health implications of the COVID-19 pandemic became apparent in 2020, the scientific community rapidly responded to an unprecedented public health threat and research challenge. Scientific projects focused on the biology of a novel coronavirus, the effects of the virus on human physiology, and the epidemiology and clinical impact of COVID-19. This knowledge was essential to the development of mitigation strategies, diagnostics, clinical care paradigms, and therapeutics to aid in the treatment of the disease and to reduce the risk of severe illness, hospitalization, and death.

During this early phase of the pandemic, very little was known about the transmission of SARS-CoV-2 infection. Those who were infected experienced a wide spectrum of symptoms, from very minor sniffles or manageable flu-like symptoms to life-threatening respiratory failure. Efforts to control the spread were hampered by a lack of information about presumed asymptomatic case rates, which made it very difficult to contain local outbreaks as these individuals were unaware if they were infected. Investigators at the National Institutes of Health (NIH) launched a study to evaluate the prevalence of immunity in the surrounding community by assaying the presence of antibodies to the virus in individuals who were not previously diagnosed with or recently experienced COVID-19 symptoms.

In a NIH press release dated April 10, 2020, Dr Anthony Fauci, Director of the NIH National Institute of Allergy and Infectious Diseases (NIAID), promoted the importance of the study [7]. Within 1 week of this announcement, NIAID received more than 400,000 emails from people across the country offering to participate in the research endeavor. The research team, which had launched the study by enrolling individuals onsite at the NIH, quickly pivoted to adapt their study design to a national-scale, remote, observational study.

**Fig. 2.**
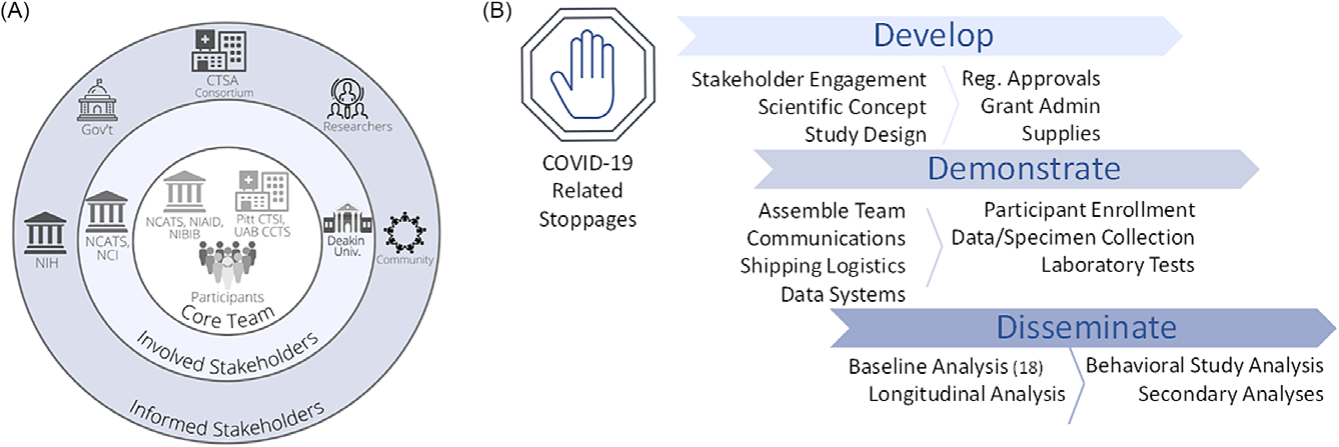
Stakeholder and study map. NIH and two NCATS-sponsored CTSA hubs (Pitt CTSI and UAB CCTS) partnered to assemble the expertise, regulatory approvals, technology, and engagement strategy to launch a national longitudinal observational seroprevalence study during the COVID-19 pandemic. A. This project engaged stakeholders on multiple levels, including project drivers involved in the concept and implementation (Core Team), sponsors (Involved Stakeholders), and several areas with vested interest (Informed Stakeholders). B. Overview of study implementation. Following widespread, COVID-19-related research stoppages that went into effect in March 2020, the team of CTSAs and NIH were able to develop the project, quickly pivoting to the scientific opportunity and scaling to a national reach. Over the next 1.5 months, the team put in place the necessary study components to allow collaborative enrollment to begin as part of the Demonstration phase. Dissemination of initial findings succeeded within ∼ 8 months. Legend: CTSA, Clinical and Translational Science Award; Gov’t, Government; NIH, National Institutes of Health; NCATS, National Center for Advancing Translational Sciences; NCI, National Cancer Institute; NIAID, National Institute of Allergy and Infectious Diseases; NIBIB, National Institute of Biomedical Imaging and Bioengineering; Pitt CTSI, University of Pittsburgh Clinical and Translational Science Institute; Reg., Regulatory; UAB CCTS, University of Alabama at Birmingham Center for Clinical and Translational Science; Univ., University

In parallel, leaders of the CTSA hubs based at the University of Pittsburgh (Pitt CTSI) and the University of Alabama at Birmingham (UAB CCTS) acknowledged that CTSAs are uniquely equipped to rapidly leverage research capacities and mobilize well-trained teams to advance clinical and translational investigation at any scale. These two CTSA hubs, which support large-scale national research projects (e.g., *All of Us* Research Program, PCORnet, ACTIV), were ideally positioned to urgently apply their experiences to assist researchers at NIAID to scale their study across the USA. This agility to engage partners to quickly and efficiently enable clinical investigation and to provide a national resource responsive to public health needs is a hallmark of CTSA programs. In 1 month, the NIAID LID Clinical Studies Unit and the Pitt and UAB CTSA hubs partnered to assemble the expertise, regulatory approvals, technology, and engagement strategy to launch a national longitudinal observational seroprevalence study during the COVID-19 pandemic (Fig. 2).

### Assembling the Team

The rapid implementation of this large, multisite, remote seroprevalence study required a transdisciplinary team of clinical investigators, laboratory scientists, biostatisticians, immunologists, infectious disease experts, epidemiologists, informaticists, clinical research coordinators, and award managers at the two CTSA hubs. This group had extensive and complementary training and skills in methodologic rigor and reproducibility, data and specimen management, regulatory knowledge, good clinical practice, and team science. The project, by design, assembled systems thinkers, communicators, domain experts, process innovators, “boundary crossers,” and team players. As a direct result of the learning opportunities available through Pitt CTSI & UAB CCTS, this team represented the characterization of translational scientists in action [8]. Additionally, given the wide range of experiences of each CTSA Hub (e.g., in supporting the *All of Us* Research Program [9]), their leadership was immediately able to plan for staffing needs, budget requirements, computing power, communication equipment, IT platforms, and space necessary for this large complex project. This shared standard of excellence in workforce development and training precedent across CTSA-based institutions was also reflected in facile communications and standard operating procedures using Salesforce Customer Relationship Management (CRM) software, Microsoft Teams, Box file storage systems, and weekly video meetings in an effort to streamline efficient and consistent support of the project at all sites.

### Regulatory Oversight

NIH served as the IRB of record to oversee all human subjects activities, adapting the original site-specific regulatory approval to a single IRB model, with Pitt CTSI and UAB CCTS establishing reliance agreements that ceded protocol review. Though the NIH IRB is not typically a resource for extramural projects, the process of rapid adaptation of site-specific protocols to shared regulatory models is a generalizable model with feasibility for any additional sites. All protocol modifications were approved by the NIH IRB (e.g., participant communications used during the recruitment, prescreening, enrollment, and sample collection phases of the project). The well-established regulatory expertise at the CTSA hubs allowed the efficient launch of the study within a few days of protocol submission and rapid approval of all amended documents throughout the project implementation. The team was also well positioned to leverage observed increases in IRB productivity and accelerated reviews of COVID human subjects protocols [10]. The synergies enabled by prior training and process know-how combined with active communication with the Office of the IRB as a partner in the process were instrumental in this accelerated time to activation.

### Study Design

Statisticians based at NIAID collaborated with colleagues in the Pitt CTSI Biostatistics, Epidemiology and Research Design (BERD) Core to revise their study design. The initial single-site recruitment model based at the NIH was insufficient for a national study design. Therefore, they adopted a quota sampling approach with algorithms to adapt recruitment targets in real time to more effectively reflect the representative diversity of the country. Their challenge, however, was how to maintain rigorous and reproducible capacities for consistent outreach, screening, enrollment, home blood sampling, and data collection of this volume.

### Information Technology Support

As part of a mission-aligned implementation strategy, CTSA organizations are developing innovative informatics solutions to use digital assets to improve health. NIH, Pitt CTSI, and UAB CCTS have established a range of expertise for the capture, management, and analysis of biological and clinical data to advance scientific discovery, following collaborative standards for interoperability and data sharing. This project used an instance of Salesforce (Salesforce, Inc.) licensed to Pitt CTSI and accessible to UAB CCTS for research purposes to track participant information, contact (both email and phone), and shipping information for the test kits. The team developed an application programming interface (API) to link Salesforce to the NIAID REDCap instance to register participants and document informed consent [11,12]. The REDCap instance was used to track consent/withdrawal status and collect patient-reported data (medical, geographic, demographic, and socioeconomic information) via electronic questionnaire. The Pitt CTSI Enterprise Relationship Management team configured the Pardot (email) and Lightning Dialer (telephone) components of Salesforce for the Pitt CTSI and UAB CCTS staff to manage the IRB-approved communication and phone outreach plans for this study. Salesforce relationship management capabilities were extended to seek and record consent from participants. Pitt CTSI wrote automated services to query the UPS and FedEx APIs, to retrieve shipping status for the kits that have gone out, and to automatically update tracking fields in Salesforce to provide a real-time picture of where participant kits were. The dedicated clinical research informatics and information technology expertise in place at the CTSA hubs and NIH allowed the facile adoption and integration of systems with the requisite security measures and rigorous data management procedures to serve a multisite clinical study.

### Participant Screening, Enrollment, Specimen, and Data Collection

This project involved quota sampling from 462,949 volunteers with the goal of enrolling a convenience sample of over 11,000 individuals reflecting demographic characteristics of the US population (see [13] for more detailed study description). The recruitment teams at Pitt CTSI and UAB CCTS followed approved telephone scripts to screen and enroll participants, which were documented in Salesforce and REDCap. Consented individuals were shipped a Mitra Home Collection Kit to provide an 80-µl blood specimen [14]. Each kit contains gauze, lancets, bandages, collection device, and all necessary shipping materials along with detailed instructions for participants and a return postage slip. Program managers at Pitt CTSI and UAB CCTS were able to package, label, and ship up to 200 kits/day. Tracking information was monitored to confirm delivery and unreturned kits, which in turn was used to trigger automated reminder messages (up to 3) and to inform the NIAID statistical team when necessary to adjust the planned consenting strategy. Follow-up email and phone messaging were facilitated by the Pitt CTSI Communications team, which also served to bolster individual participant understanding when needed (e.g., how to use the collection kit). Specimen kits were returned directly to the NIH for laboratory analysis.

Participant-reported health data and personality survey feedback were captured digitally using an emailed link to a REDCap questionnaire or facilitated by a study team member by telephone if necessary. While the project’s screening, recruitment, and collection components were customized to the study aims and did not exist “on the shelf,” the team was able to draw on experience in multimodal communication methods, near-peer mentoring, and design thinking – common topics in CTSA-based workforce development programs – to rapidly prototype and refine solutions to overcome barriers to shipping kits, calling participants in different time zones and other logistical challenges.

### Research Dissemination

The vested interest of the public to participate in this seroprevalence study and to contribute to the understanding of a disease that affected everyone’s lives is exemplified by the initial response of individuals volunteering to participate in the study within hours of NIAID’s press release. While return of individual results was not possible, the team was committed to scientific transparency and, therefore, acquired regulatory approval to share summary data with participants by emailing them the initial published, peer-reviewed manuscript of baseline study results [13]. Similarly, understanding the urgency to share COVID-19 results, the data were published on the medRxiv preprint server a few months earlier for rapid access by the scientific community [15,16].

## Discussion, Lessons Learned, and Perspectives for the Future

**Fig. 3.**
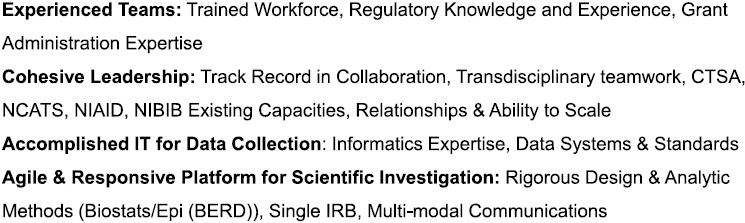
Key advantages for rapid response. Legend: CTSA, Clinical and Translational Science Award; IRB, Institutional Review Board; IT, Information Technology; NCATS, National Center for Advancing Translational Sciences; NCI, National Cancer Institute; NIAID, National Institute of Allergy and Infectious Diseases; NIBIB, National Institute of Biomedical Imaging and Bioengineering.

Rapidly launching a large, multisite, decentralized clinical research study to enroll participants across the nation and collect biospecimens and data is a complex endeavor, even under traditional circumstances. Carrying out the study in the context of a global health crisis that led to hiring freezes and furloughs of research staff, virtual (socially distanced) work, and supply chain constraints added to the project management’s complexity. Yet, as is apparent by the present study, CTSA hubs are optimally positioned to rapidly implement high-impact translational research projects at local, regional, and national levels, even under the most challenging circumstances, as a direct result of their expertise, research capacities, infrastructure, and established collaborations established years before the pandemic (Fig. 3).

## CTSA Advantage for Large-Scale, Rapid Deployment

### Transdisciplinary Teamwork

The advancement of scientific discovery to improve health is increasingly team science. The ability for diverse investigative teams to work together toward a shared goal is a strength in the conduct of science [17]. Sponsorship of this project relied on intra-agency collaboration at the NIH, bringing together NIAID, NCATS, NIBIB, and NCI to support the project. Conduct of the project relied on seamless integration of daily activities across NIH (NCATS, NIAID LID CSU, NIBIB) and CTSA organizations (Pitt CTSI and UAB CCTS). The team – comprised of faculty, students, and staff – represented clinical investigators, laboratory scientists, biostatisticians, epidemiologists, health economists, informaticists, data managers, research coordinators, and more. The success of this multidisciplinary team to rapidly launch and complete a high-impact study on a national scale is a direct result of each member’s training, skills, and experience in core principles of clinical and translational team science.

### Translational Spectrum

The seroprevalence study collaboration integrated all phases of the translational research spectrum, which are supported by CTSA hubs [18]:

T1 – Basic Research: Use of ELISA to detect SARS-CoV-2-related antibodies in blood

T2 – Clinical Research: National population enrollment and specimen collection

T3 – Population Research: Outcomes investigation of seropositivity relative to gender, race/ethnicity, urban/rural, geography, etc.

T4 – Public Health Research: Value of seropositivity estimates in guiding pandemic response and understanding regional variability

### Agile and Responsive Research Capacity

Like the NIH, the CTSA consortium is part of a national resource committed to the development of scientific and operational innovations that improve the efficiency and effectiveness of clinical and translational investigations while maintaining high standards of research quality, reproducibility, and safety. The CTSA hubs are highly adept at promoting partnerships and collaborations to accelerate these processes and in developing a diverse, skilled, and knowledgeable workforce capable of supporting the life cycle of a study. Accordingly, the hubs based at Pitt CTSI and UAB CCTS were able to rapidly respond to the call to expand a local project from NIH to a national scale [19]. They leveraged their experiences with large research programs like *All of Us* and PCORnet to adapt to the tailored needs of the new project. They had well-established infrastructure, relationships, and expertise that could guide the startup and conduct of the project within each site and the communication platforms to harmonize standard operating procedures across sites. The similarities in training between CTSA hubs also allowed team members to relate, to mentor, and to collaborate with each other in support of the project. This cross-coverage between sites was especially helpful in both grants management and regulatory approvals as well as managing participant outreach to different time zones and appreciating variable work preferences (early risers and night owls).

While the study team was expeditious and flexible in implementation, the speed of the pandemic spread proved challenging. The rate of transmission, mutation rate of the virus, development and deployment of vaccines, and the adaptive sampling strategy necessitated considerations in data collection and rigorous analysis that delayed the availability of study insights to impact COVID-19 public health policy and management. While some other population-based serosurveys conducted in the USA were published more quickly [20-28] or represented larger enrollments [26,29,30], they were all limited to a constrained geographic region (e.g., city, county/parish, state). This seroprevalence experiment is the only known project conducted on a national scale, recruiting over 11,000 individuals and publishing baseline data within 10 months of the first enrollment in March 2020 (Fig. 1) [13]. The experience and established methods will be exceptionally valuable to future pandemic preparedness and response other health crises.

## Conclusion – Preparing for the Future

Response to scientific opportunity, whether prompted by a global pandemic or not, requires greater speed and efficiency to launch studies, recruit participants, analyze data, and disseminate results. As evidenced in this use case, institutions and investigators are better equipped to address emerging research needs in an environment that has assembled resource components (informatics/IT, cohesive leadership, biostatistics, trained workforce, grants management, regulatory expertise/good clinical practice, etc.) and a track record in external partnerships. Experience with verbal informed consent, digital survey of participant information, and remote sample collection can yield greater enrollments, reach remote (e.g., rural) geographic areas, and increase the diversity and inclusion of groups underrepresented in biomedical research. The involvement of multiple sites expands this functionality and offers an understanding of cultural sensitivities, health literacy, and local attitudes. Challenges of harmonizing activities in multiple locations can be minimized with a proactive training, communication, and capacity building. This seroprevalence study is an important demonstration of how the complementary capabilities of CTSA hubs can come together to achieve programmatic synergies and rapidly conduct large-scale studies on a national level, an experience that will inform and enable collaborations and pandemic preparedness in the future.

## References

[1] World Health Organization. WHO Director-General’s opening remarks at the media briefing on COVID-19, 2020. (https://www.who.int/dg/speeches/detail/who-director-general-s-opening-remarks-at-the-media-briefing-on-covid-19---11-march-2020)

[2] History. Com Editors. First confirmed case of COVID-19 found in U.S., 2020. (https://www.history.com/this-day-in-history/first-confirmed-case-of-coronavirus-found-in-us-washington-state)

[3] Elflein J. Number of cumulative cases of coronavirus (COVID-19) worldwide from January 22, 2020 to April 10, 2022, by day, 2022. (https://www.statista.com/statistics/1103040/cumulative-coronavirus-covid19-cases-number-worldwide-by-day/)

[4] The New York Times. Coronavirus in the U.S.: Latest Map and Case Count, 2022. (https://www.nytimes.com/interactive/2021/us/covid-cases.html)

[5] Zerhouni E. Translational and clinical science--time for a new vision. New England Journal of Medicine 2005; 353(15): 1621–1623. DOI 10.1056/NEJMsb053723. 16221788

[6] Austin C. Opportunities and challenges in translational science. Clinical and Translational Science 2021; 14(5): 1629–1647. DOI 10.1111/cts.13055. 33982407 PMC8504824

[7] National Institutes of Health. NIH begins study to quantify undetected cases of coronavirus infection, 2020. (https://www.nih.gov/news-events/news-releases/nih-begins-study-quantify-undetected-cases-coronavirus-infection)

[8] Gilliland CT , White J , Gee B , et al. The fundamental characteristics of a translational scientist. ACS Pharmacology & Translational Science 2019; 2(3): 213–216. DOI 10.1021/acsptsci.9b00022. 32259057 PMC7088880

[9] All of Us Research Program Investigators, Denny JC , Rutter JL , et al. The “All of Us” Research Program. New England Journal of Medicine 2019; 381(7): 668–676. DOI 10.1056/NEJMsr1809937. 31412182 PMC8291101

[10] Ford DE , Johnson A , Nichols JJ , Rothwell E , Dubinett S , Naeim A. Challenges and lessons learned for institutional review board procedures during the COVID-19 pandemic. Journal of Clinical and Translational Science 2021; 5(1): e107. DOI 10.1017/cts.2021.27. 34192061 PMC8220014

[11] Harris PA , Taylor R , Thielke R , Payne J , Gonzalez N , Conde JG. Research electronic data capture (REDCap)--a metadata-driven methodology and workflow process for providing translational research informatics support. Journal of Biomedical Informatics 2009; 42(2): 377–381. DOI 10.1016/j.jbi.2008.08.010. 18929686 PMC2700030

[12] Harris PA , Taylor R , Minor BL , et al. The REDCap consortium: building an international community of software platform partners. Journal of Biomedical Informatics 2019; 95: 103208. DOI 10.1016/j.jbi.2019.103208. 31078660 PMC7254481

[13] Kalish H , Klumpp-Thomas C , Hunsberger S , et al. Undiagnosed SARS-CoV-2 seropositivity during the first 6 months of the COVID-19 pandemic in the United States. Science Translational Medicine 2021; 13(601): eabh3826. DOI 10.1126/scitranslmed.abh3826. 34158410 PMC8432952

[14] Hall EW , Luisi N , Zlotorzynska M , et al. Willingness to use home collection methods to provide specimens for SARS-CoV-2/COVID-19 research: survey study. Journal of Medical Internet Research 2020; 22(9): e19471. DOI 10.2196/19471. 32790639 PMC7473702

[15] Kalish H , Klumpp-Thomas C , Hunsberger S , et al. Mapping a pandemic: SARS-CoV-2 Seropositivity in the United States. 2021, 10.1101/2021.01.27.21250570 Preprint. medRxiv. 2021;2021.01.27.21250570.

[16] Else H. How a torrent of COVID science changed research publishing - in seven charts. Nature 2020; 588(7839): 553–553. DOI 10.1038/d41586-020-03564-y. 33328621

[17] Science benefits from diversity. Nature 2018; 558(7708): 5. DOI 10.1038/d41586-018-05326-3. 31076730

[18] National Center for Advancing Translational Sciences. Translational Science Spectrum, 2021. (https://ncats.nih.gov/translation/spectrum)

[19] Austin CP , Jonson S , Kurilla MG. Foreword to the JCTS COVID-19 special issue. Journal of Clinical and Translational Science 2021; 5(1): e103. DOI 10.1017/cts.2021.400. 34164155 PMC8190713

[20] Barzin A , Schmitz JL , Rosin S , et al. SARS-CoV-2 seroprevalence among a Southern U.S. population indicates limited asymptomatic spread under physical distancing measures. mBio 2020; 11(5): e02426–e02420. DOI 10.1128/mBio.02426-20. 32994333 PMC7527736

[21] Dingens AS , Crawford KHD , Adler A , et al. Serological identification of SARS-CoV-2 infections among children visiting a hospital during the initial Seattle outbreak. Nature Communications 2020; 11(1): 4378. DOI 10.1038/s41467-020-18178-1. PMC7463158 32873791

[22] Feehan AK , Fort D , Garcia-Diaz J , et al. Seroprevalence of SARS-CoV-2 and infection fatality ratio, Orleans and Jefferson Parishes, Louisiana, USA, May 2020. Emerging Infectious Diseases 2020; 26(11): 2766–2769. DOI 10.3201/eid2611.203029. 32731911 PMC7588526

[23] Menachemi N , Yiannoutsos CT , Dixon BE , et al. Population point prevalence of SARS-CoV-2 infection based on a Statewide Random Sample - Indiana, April 25-29, 2020 [published correction appears in MMWR Morb Mortal Wkly Rep. 2020 Aug 14;69(32):1106]. MMWR. Morbidity and Mortality Weekly Report 2020; 69(29): 960–964. DOI 10.15585/mmwr.mm6929e1. 32701938 PMC7377824

[24] Ng DL , Goldgof GM , Shy BR , et al. SARS-CoV-2 seroprevalence and neutralizing activity in donor and patient blood. Nature Communications 2020; 11(1): 4698. DOI 10.1038/s41467-020-18468-8. PMC7499171 32943630

[25] Ripperger TJ , Uhrlaub JL , Watanabe M , et al. Orthogonal SARS-CoV-2 serological assays enable surveillance of low-prevalence communities and reveal durable humoral immunity. Immunity 2020; 53(5): 925–933.e4. DOI 10.1016/j.immuni.2020.10.004. 33129373 PMC7554472

[26] Rosenberg ES , Tesoriero JM , Rosenthal EM , et al. Cumulative incidence and diagnosis of SARS-CoV-2 infection in New York. Annals of Epidemiology 2020; 48: 23–29.e4. DOI 10.1016/j.annepidem.2020.06.004. 32648546 PMC7297691

[27] Sood N , Simon P , Ebner P , et al. Seroprevalence of SARS-CoV-2-specific antibodies among adults in Los Angeles County, California, on April 10-11, 2020. JAMA 2020; 323(23): 2425–2427. DOI 10.1001/jama.2020.8279. 32421144 PMC7235907

[28] Sutton M , Cieslak P , Linder M. Notes from the field: seroprevalence estimates of SARS-CoV-2 infection in convenience sample - Oregon, May 11-June 15, 2020. MMWR. Morbidity and Mortality Weekly Report 2020; 69(32): 1100–1101. DOI 10.15585/mmwr.mm6932a4. 32790658 PMC7440123

[29] DeSantis SM , León-Novelo LG , Swartz MD , et al. Methodology to estimate natural- and vaccine-induced antibodies to SARS-CoV-2 in a large geographic region. PLoS One 2022; 17(9): e0273694. DOI 10.1371/journal.pone.0273694. 36084125 PMC9462720

[30] Pathela P , Crawley A , Weiss D , et al. Seroprevalence of severe acute respiratory syndrome Coronavirus 2 following the largest initial epidemic wave in the United States: findings from New York City, 13 May to 21 July 2020. Journal of Infectious Diseases 2021; 224(2): 196–206. DOI 10.1093/infdis/jiab200. 33836067 PMC8083309

